# The combination matters - distinct impact of lifestyle factors on sperm quality: a study on semen analysis of 1683 patients according to MSOME criteria

**DOI:** 10.1186/1477-7827-10-115

**Published:** 2012-12-24

**Authors:** Johannes Wogatzky, Barbara Wirleitner, Astrid Stecher, Pierre Vanderzwalmen, Anton Neyer, Dietmar Spitzer, Maximilian Schuff, Birgit Schechinger, Nicolas H Zech

**Affiliations:** 1 IVF Centers Prof Zech-Bregenz, Roemerstrasse 2, Bregenz, 6900, Austria; 2 Centre Hospitalier Inter Régional Cavell (CHIREC), 1420 Braine-l‘alleud, Brussels, Belgium; 3 IVF Centers Prof Zech-Salzburg, Innsbrucker Bundesstr. 35, Salzburg, 5020, Austria

**Keywords:** Sperm quality, MSOME, IMSI, Lifestyle, BMI, Ejaculation frequency, Oxidative stress

## Abstract

**Background:**

Poor sperm quality can negatively affect embryonic development and IVF outcome. This study is aimed at investigating the influence of various lifestyle factors on semen quality according to MSOME (motile sperm organelle morphology examination) criteria.

**Methods:**

1683 male patients undergoing assisted reproductive technologies (ART) in our clinic were surveyed about their age, BMI (body mass index), ejaculation frequency, nutrition, sports, sleeping habits and social behavior. Semen samples were collected and evaluation of semen parameters according to MSOME and WHO criteria was performed. Results were grouped and statistically analyzed.

**Results:**

Although single parameters had minor effects on sperm parameter, the combination of age, BMI, coffee intake, ejaculatory frequency and duration of sexual abstinence were identified as factors having a negative effect on sperm motility. Additionally, we could demonstrate that MSOME quality was reduced. The negative impact of age, BMI and coffee intake on sperm quality could be compensated if patients had a high ejaculation frequency and shorter periods of sexual abstinence.

**Conclusions:**

Combinations of adverse lifestyle factors could have a detrimental impact on sperm, not only in terms of motility and sperm count but also in terms of sperm head vacuolization. This negative impact was shown to be compensated by higher ejaculation frequency and a shorter period of sexual abstinence. The compensation is most likely due to a shorter storage time in the male gonads, thus reducing the duration of sperms’ exposure to reactive oxygen species (ROS).

## Background

It is increasingly accepted that lifestyle factors have an impact on sperm quality. Among the individual factors which are considered to increase or decrease sperm quality in humans as defined by the Kruger or WHO (World Health Organization) criteria [1-5] are environment, occupation, nutrition, stimulants, ejaculation frequency and lifestyle choices. Motility, sperm count, sperm size and shape are routinely analyzed during in vitro fertilization/ intracytoplasmic sperm injection (IVF/ICSI).

Semen parameters are evaluated by different criteria, some of which are given by Kruger [6], the Tygerberg criteria [7] and the WHO [8,9]. Owing to the improvement of the technical facilities in the IVF-laboratories, new possibilities have arisen and we are now able to analyze the sperm head in more detail. The introduction of MSOME (Motile Sperm Organelle Morphology Examination) by Bartoov and colleagues [10] permits the examination of subcellular defects like nuclear vacuoles at high magnification (6000-12500x) in real time on vital sperm. Such defects are not detectable under standard magnification during ICSI [10,11]. The application of the MSOME-approach in ART-cycles has demonstrated to significantly improve implantation and pregnancy rates and to reduce the miscarriage rates [10,12-18]. Therefore, MSOME has been considered as representing an improvement in the evaluation of semen quality [19,20]. MSOME that was subsequently applied to complement ICSI and IMSI (Intracytoplasmic Morphologically Selected Sperm Injection) then became a standard technique in assisted reproduction.

The origin of the nuclear vacuoles detected by MSOME is still under debate. Nevertheless, several recent studies demonstrated that nuclear vacuoles were correlated to DNA damage and/or failures in chromatin packaging [20-23]. Sperm chromatin packaging and DNA integrity are crucial for good sperm quality as DNA damage has been found to impair reproductive outcomes due to the late paternal effect [24,25]. In addition, spermatozoa from infertile men were shown to present markedly more DNA damage than sperms from fertile men [26].

It has to be considered that the patient collective undergoing ART cannot be compared to the fertile population. Therefore, defining impact factors acting on sperm quality in this specific subgroup may differ from impact factors in healthy men. Thus, we investigated different lifestyle factors, that beside nutrition, smoking and alcohol consumption included factors such as age, BMI (body mass index), coffee consumption, sexual abstinence and ejaculatory frequency. The objective of the present study was to elucidate the influence of multiple personal and lifestyle factors on sperm quality according to MSOME criteria in a group of men attending a fertility clinic.

## Methods

### Study participants

Our study aimed to investigate a putative influence of lifestyle factors on sperm quality according to WHO as well as MSOME criteria employing an extensive questionnaire. 1683 men undergoing ART at our center filled in this questionnaire answering questions regarding age, BMI, caffeine and alcohol consumption, smoking, exercise, sauna, frequency of sexual activity, possible increased stress-perception and nutrition behavior. The patients participating in the study were recruited according to following criteria: they shall not have any known genetic reasons for an impaired spermiogram such as Klinefelter syndrome (XXY condition) or shall not present any deletions of AZF regions. Moreover, all men with abnormal sperm sample were advised to get a urological workup to rule out a varicocele, abnormal testicular volume or even testicular cancer and were excluded from this study. The written informed consent was obtained from all participants of this study.

### Determination of sperm morphology by MSOME

Analysis of spermatozoa was performed at 6000x magnification under a Nomarski interferential Leica AM 6000 inverted microscope (Leica, Germany). Classification in three categories was done according to Vanderzwalmen et al., 2008 [12] for every sperm sample. MSOME involves the grading of spermatozoa at 6000x magnification according to the presence of nuclear vacuoles:

grade I, normal shaped sperm without vacuoles or with 1–2 small vacuoles < 4% of the head length.

grade II, normal shaped sperm with one or more large vacuoles > 4% head area.

grade III, sperm with abnormal morphology with our without vacuoles.

Sperm count and motility were assessed according to WHO criteria [9].

### Statistical evaluation

Statistical analysis was performed using the Statistical Program for Social Science (SPSS 13.0, Chicago, IL) software. Continuous variables were reported as the mean ± standard deviation (s.d.) and range. Between-group comparisons of normally distributed variables were assessed using Student's *t*-test. Data were retrospectively correlated to their conventional semen analysis and MSOME results. For multifactorial lifestyle influence patients were evaluated with a point-based system with a cut-off value >2 for ‘unhealthy’ lifestyle. Between-group comparisons were assessed using Student's *t*-test.

## Results

### Patient characteristics

The study population completing the questionnaire comprised a total of 1683 male patients who were being treated at our fertility clinic. Patients’ characteristics and lifestyle behavior were summarized in Table 1 and 2, and see Additional file 1: Table S1. Semen characteristics of all patients were presented as mean ± s.d. (see Table 3).

**Table 1:** Population and lifestyle characteristics of patients

	**Number of patients**	**Mean ± s.d.**	**Range**
*Total number of patients*	1683		
*Mean age (years)*	1683	40.4 ± 5.9	25-72
*BMI (kg/m*^*2*^*)*	1678	26.1± 3.4	17-48
*Period of sexual abstinence (days)*	1675	3.2 ± 2.2	0-35
*Ejaculatory frequency (ejaculations per month)*	1658	6.7 ± 3.4	0-24
*Working hours (h)*	1589	9.4 ± 1.4	0-15
*Sleeping/day (h)*	1591	7.1 ± 0.8	4-12
*Coffee intake (cups/day)*	1321	2.0 ± 1.5	0-12
*Meals intake/day*	1564	2.7 ± 0.6	1-5
*Fruit/vegetables intake (portion/day)*	1499	1.3 ± 0.8	0-5
*Fluid intake (liter/day)*	1569	2.1 ± 0.7	0.5-6
*Smoking: Yes (Range: cigarettes/day)*	215/1438 ^a^	13 ± 8.4	0.5-50 ^b^
*Endurance sports (h/week)*	899/1454 ^a^	2.6 ± 2.1	0.5-22
*Endurance sports (times/week)*	712/1421 ^a^	2.3 ± 1.5	0.5-14
*Athletic sports (h/week)*	286/1514 ^a^	2.4 ± 1.2	0.5-14
*Athletic sports (times/week)*	275/1505 ^a^	2.1 ± 1.1	0.5-7

Values were expressed as mean ± s.d. (standard deviation).

Number of patients in this group/patients answered this question. ^a^ Number of patients affirmed this question. ^b^ cigarettes/ day.

**Table 2:** Population and lifestyle characteristics of patients

		**Number of patients answered**	**Number of patients affirmed this question**	**Percentage (%) of total patients**	**Casually**	**Regularly**
*Total number of patients*		1683		100		
*Fever during the last 3 months*		1579	152	9.0		
*Mumps*		1595	262	15.6		
*Clamydia infection*		1603	40	2.4		
*Operation*		1607	162	9.6		
*Operation of groin area*		1608	131	7.8		
*Chemotherapy*		1606	11	0.7		
*Pain*		1592	110	6.5		
*Allergy*		1588	516	30.7		
*No family pre-existing conditions*^*a*^		1601	984	58.5		
*Drug intake*		1577	422	25.1		
*Vitamin intake*		1579	849	50.5		
*Healthy or very healthy nutrition according to self-assessment*		1567	1532	91.0		
*No regular meal intake*		1571	183	10.9		
*Diet*		1569	52	3.1		
*Alcohol intake*		1575	1294	76.9	1127 (67.0%)	167 (9.9%)
* Rather beer*		1238	256	15.2		
* Rather red wine*		1237	165	9.8		
*Coffee intake*		1498	1321	78.5		
*Smoking*		1575	354	21.0	155 (9.2%)	199 (11.8%)
*Stress*		1590	1397	83.0		
*regular sleep/wake cycle*		1547	1361	80.9		
*Sport*		1244	1215	72.2		
* Medicine for output increase*		1576	63	3.7		
* Endurance sports*		1563	899	53.4		
* Athletic sports*		1562	340	20.2		
*Sauna (regularly)*		1576	153	9.1		

Values were expressed as percentage. ^a^ Pre-existing conditions such as cancer diabetes; Morbus Alzheimer.

**Table 3:** Semen characteristics of patients

**Sperm parameter**		**Mean ± s.d.**	**Range**
*Ejaculation volume (ml)*		2.7 ± 1.5	0.2 - 9.0
*Sperm concentration (Mio/ml)*		23.4 ± 29.2	0.0001 - 424
*Total sperm count (TSC)*		58.8 ± 92.9	0.001 - 2077
*Motility (according to WHO criteria) %*	*Grade a*	4.8 ± 7.9	0 - 70.0
	*Grade b*	31.9 ± 17.7	0 - 100
	*Grade c*	18.0 ± 14.0	0 - 100
	*Grade d*	45.3 ± 17.4	0 - 100
*Morphology (according to MSOME criteria) %*	*Class I*	6.1 ± 6.3	0 - 45
	*Class II*	43.3 ± 14.4	0 - 78
	*Class III*	50.6 ± 17.8	0 - 100

Semen characteristics of the total number of patients. Values were expressed as percent or average ± s.d.

The demographic characteristics were as follows (mean ±): age, 40.4 ± 5.9 years; BMI, 26.1 ± 3.4 kg/m^2^. The mean period of abstinence and the mean ejaculation frequency was 3.2 days ± 2.2 and 6.7 times per month ± 3.4, respectively.

In respect to the possibility that putative pre-conditions might affect patients’ spermatogenesis, we asked them about diseases and operations. 152 patients stated that they had fever within the last 3 month, 262 men have had *parotitis epidemica* before*,* and 40 participants have suffered from a *clamydia trachomatis* infection in the past. 162 patients had undergone an operation and 131 of them in particular an operation in the groin area. Moreover, 11 patients have already undergone chemotherapy. 516 patients declared to be suffering from allergies and 110 stated that at the moment, they are in pain due to different reasons (Table 2). 422 patients have taken or are still taking drugs, most of them painkillers or antibiotics (Table 1 and 2, see Additional file 1: Table S1).

### Influence of specific lifestyle factors on semen quality

Quality and quantity of food intake is supposed to influence semen parameters [1]. In the first place, we asked patients for a self-assessment of their dietary status. Interestingly, 91% of all patients questioned (n= 1532) assumed that their nutrition was healthy or very healthy. Only 33 men (2%) considered their diet as unhealthy (junk-food). In contrast, when asked how many servings of fruit and vegetables were consumed per day on average, an astonishing 62.4% (n=1050) of them had only one or no serving of vegetables or fruit (average value regarding the total number of participating patients: 1.3 ± 0.8) a day. Even though, poor fruit and vegetable intake did not correlate significantly with sperm quality in this study, probably due to the specific subgroup of patients mentioned above, it is widely accepted, that healthy nutrition is supposed to have an important impact on sperm quality of healthy men.

Additionally, regarding their body mass index (BMI), the average of patients ranged in between BMI 25–30 kg/m^2^ (Table 1), so in within the pre-obese (moderately obese) according to World Health Organization BMI classification [27]. In concordance to these observations, only 52 men declared to have dieted before (3.1%).

Over- and underweight is seen as a risk factor for low sperm quality [2,28-30]. Assessing the possible relationship between BMI and sperm quality, we found significantly less immotile sperms in men with BMI <25 (n=692) versus BMI >25 (n=986), (p<0.05) (Table 4). In addition, less sperms and fewer class I spermatozoa according to MSOME criteria were detected in the (pre-) obese group, although not significant.

**Table 4:** Lifestyle factors (BMI, age, caffeine consumption, sexual behavior, smoking and stress linked to analyzed semen parameters

		**Number of patients**	**Ejaculation volume (ml/semen sample)**	**Sperm concen-tration Mio/ml**	**TSC Mio/ sample**	**Motility**				**MSOME**		
						**Grade a**	**Grade b**	**Grade c**	**Grade d**	**Class I**	**Class II**	**Class III**
*BMI (kg/m*^*2*^*)*	<25	692	2.8 (1.4)	24.3 (31.2)	63.2 (94.7)	5.1 (7.9)	32.8 (17.6)	18.6 (14.3)	43.5 ^a^ (20.6)	6.4 (6.6)	43.3 (14.2)	50.3 (17.8)
	>25	986	2.6 (1.5)	22.5 (26.3)	55.2 (90.8)	4.7 (7.9)	31.4 (17.8)	17.7 (13.9)	46.2 (20.7)	5.8 (6.1)	43.2 (14.6)	51 (17.9)
*Age (years)*	<50	1598	2.7 (1.5) ^c^	23.5 (29.6)	59.8 (94.7) ^b^	5.0 (7.9) ^b^	32.2 (17.7) ^b^	18,2 (14.1)	44.6 ^c^ (20.6)	6.1 (6.4)	43.2 (14.5)	50.7 (17.9)
	>50	85	2.0 (1.2)	22.1 (19.3)	41.7 (43.5)	2.9 (6.9)	26.7 (16.5)	17.7 (12.3)	52.7 (20.4)	5.4 (5.1)	44.8 (12.5)	49.8 (15.7)
*Coffee cups/day*	<3	1479	2.7 (1.5)	23.1 (28.9)	58 (91.2)	4.9 (7.9)	32.1 (17.8)	17.9 (14.1)	45.1 (20.6)	6.2 (6.4)	43 (14.3)	50.8 (17.8)
	>3	204	2.6 (1.5)	25.8 (31.5)	65.1 (104.1)	4.3 (8.1)	30.9 (16.7)	18.2 (12.9)	46.6 (21.4)	5.5 (5.6)	45.3 (15.2)	49.2 (18.0)
*Abstinence period (days)*	<2	649	2.2 ^b^ (1.2)	23.2 (33.2)	48.7 ^c^ (111.9)	4.8 (7.9)	31.4 (18.4)	18.8 (14.9)	45.0 (20.8)	6.4 (6.5)	42.8 (14.5)	50.8 (18.3)
	>2	1033	3 (1.5)	23.7 (26.4)	65.3 (78.1)	4.9 (7.9)	32.3 (17.3)	17.4 (13.4)	45.4 (20.6)	5.8 (6.2)	43.5 (14.3)	50.7 (17.5)
*Ejaculation frequency times/month*	>4	1519	2.7 (1.4)	23.4 (29.8)	58.4 (95.3)	4.9 (8.0)	32.2 (17.6)	18 (14)	44.9 (20.4)	6.2 (6.4) ^a^	43.3 (14.5)	50.5 (18.0)
	<4	140	2.9 (1.6)	23.5 (23.5)	63.5 (66.9)	4.7 (7.0)	30.3 (18.5)	17.8 (14.8)	47.2 (22.9)	5.1 (5.5)	43.1 (13.2)	51.8 (15.9)
*Alcohol intake*	No	282	2.8 (1.4)	21.3	57.4	5.0	32.7	16.0	46.3	5.7	43.0	51.3
	occasionally	1127	2.7 (1.4)	23.5	59.3	4.7	32.0	18.4	44.9	6.1	43.1	50.8
	frequent	164	2.7	26.7	61.9	5.0	32.3	18.2	44.5	6.3	45.0	48.7
*Smoking*	No/ occasionally	1380	2.7	23.5	59.8	4.8	31.9	17.9	45.4	6.1	42.9 ^b^	51.0 ^a^
	frequent	199	2.5	22.5	54.7	5.1	33.7	16.8	44.4	6.1	45.7	48.2
*Stress*	No	1163	2.7	23.0	58.0	4.6 ^a^	31.4 ^b^	18.1 ^b^	45.9	5.9 ^a^	42.6 ^b^	51.5 ^b^
	Yes	431	2.7	24.4	62.5	5.5	34.1	17.2	43.2	6.6	45.0	48.4

BMI: body mass index; TSC: total sperm count; standard deviation is given in brackets, ^a^ P value <0.05; ^b^ P value < 0.005; ^c^ P value < 0.001.

Does age have an influence on semen quality? Several reports have claimed an inverse correlation between age and sperm motility. Furthermore, a recent study by Silva et al., [31] has postulated a correlation between advanced age and poor sperm morphology according to MSOME criteria, as shown with 975 men undergoing evaluation or fertility treatment. In concordance to these findings, it could be further demonstrated by several studies that advanced paternal age results in a higher risk of schizophrenia, autism, bipolar disorders or impaired neurocognitive capability in their offspring [32-35], indicating that sperm quality substantially decreases during the aging process.

Mean age of our male patients was 40.4 ± 5.9 years. We saw a significant decrease in the ejaculation volume and semen quality regarding the total sperm count (TSC) and the motility in older patients (age >50 years, n=85) compared to younger patients (age <50, n=1598). Moreover, in terms of morphology, the percentage of class I sperm declined perspicuously, although not significantly (see Table 4).

In our questionnaire we additionally asked the patients about their sexual behavior with reference to their ejaculation frequency and period of sexual abstinence. High turn-over of spermatozoa might protect them from senescence or long-term exposure to reactive oxygen species (ROS), generated by leucocytes or other toxic substances. Correlations between sperm quality and ejaculation were e.g. found by Oldereid and colleagues [36], reporting a significant increase in sperm motility as a result of a higher ejaculation frequency.

1033 patients stated that they have practiced sexual abstinence for more than 2 days. In this patient subgroup we observed significantly higher ejaculation volumes and total sperm counts as expected. Although we found a higher percentage of class I spermatozoa when the abstinence was shorter than 2 days (6.4 vs. 5.8%), the difference was statistically not significant. However, regarding the ejaculation frequency, we observed a significantly better sperm morphology (6.2 vs. 5.1% class I sperm) when patients stated to have more than 4 ejaculations per month (Table 4).

Stimulants such as alcohol, tobacco and coffee were predominantly considered as sperm damaging substances. Several studies postulated adverse effects of high caffeine consumption for semen quality. In a recent study by Jensen and colleagues, a correlation of high caffeine intake and reduced sperm concentration as well as total sperm count has been found [4]. In our study 78.5% (1321 men) admitted coffee consumption (Table 2). 204 men out of these revealed a coffee intake of more than 3 cups of coffee per day (Table 4). With respect to MSOME criteria, these patients revealed a marked tendency towards lower sperm quality (Table 4). Surprisingly, no effect from alcohol consumption according to MSOME or WHO criteria could be found in our patient collective. Patients were grouped according to their stated drinking behavior into non-alcohol consumers (n=282), occasional alcohol consumers (n=1127) and frequent consumers (n=164). No significant differences in semen parameters between these 3 subgroups were found. Although it is widely accepted that ethanol and its metabolized products are cell-toxic, it should also be kept in mind that wine or beer contain polyphenoles such as resveratrol or xanthohuminol, which were demonstrated to have a strong therapeutic and cell-protective potential. However, even regarding their favorite alcoholic drink (256 stated beer as favored drink, 165 said to prefer red wine), we could find no difference (data not shown). The same was observed for general intake of non-alcoholic drinks (tea, water, juice)/day. We could establish no difference whether the men drank 1 – 2 or 3 liters of fluids per day (data not shown).

Nicotine abuse is also considered to harm spermatozoa by changing blood circulation, hormone levels, increasing oxidative stress, promoting the accumulation of DNA-benzopyrene adducts and cadmium, DNA fragmentation and chromosomal aberrations. Several publications indicate adverse effects of smoking on sperm parameters [1,37-39]. We therefore asked patients about their smoking habits. According to the patient’s questionnaire 1380 patients were non-smokers and 354 smokers. 199 (49.2%) of them admitted frequent smoking. Assuming that continuous exposure of sperm to smoke-related toxins may affect their quality, we compared semen parameters of frequent smokers to those of non- and/or occasional smokers. Astonishingly enough, we found no serious differences in total sperm count, sperm concentration and motility, but a significant shift in sperm morphology from class III to class II (see Table 4), due to unknown reasons. Even when we factored the quantity of consumed cigarettes per day, we found almost the same results (data not shown). The interpretation of these findings is difficult, but could be attributable to the fact, that couples who have to undergo ART will adapt their lifestyle also on the grounds of their unwanted childlessness. Because smoking is widely known as impact factor on sperm quality, this will lead to lifestyle modifications. Knowing you have poor sperm quality will more likely result in quitting smoking than knowing about excellent sperm parameters and a blocked fallopian tube of the partner. Therefore correlating smoking habits retrospectively to sperm quality in this subpopulation is challenging.

Mental illness such as burnout and depression is considered to be a steadily rising epidemic due to our modern urban-industrial fast-paced work environment and western lifestyle associated with increased job strain. Additionally, the undesired childlessness of IVF patients and increasing pressure of expectations might have further detrimental impacts on semen quality due to hormonal alterations. We therefore asked our patients about their psychological status. In patients reporting severe stress (n=431) we found - in comparison to the remainder of patients (n=1163) -, quite astonishingly, significantly more motile sperm (grade a and b) in the “stressed group”. Furthermore, we also observed a significant improvement in sperm morphology according to MSOME criteria (6.6% class I vs. 5.9%). Although it is commonly accepted from the animal model systems that stress is detrimental for fertility, the outcome of studies regarding humans is quite controversial [40-43]. It might therefore be crucial to focus on the causation and the duration of emotional pressure and to distinguish between eustress (positive stress) and distress (negative stress). Since we have no further information regarding these aspects, further explanation attempts might only be speculative.

Shift work can cause circadian disorder, insomnia and may affect general health and is moreover harmful to fertility [44]. Surprisingly, with respect to the rapid augmentation of shift work in Central Europe during the last decades and the assumed mental pressure, still 88% of all our patients stated to have regular sleep/wake cycles. An impact on sperm parameters could not be found.

Regular physical exercise can reduce BMI, alternate hormone levels in a positive manner and enhance the libido. As regards physical exercise, 72.2% (n=1215) of our patients reported occasional aerobic exercise such as jogging, walking or cycling or others and even 38.5% (n=397) reported to exercise frequently. 153 men stated at least one sauna visit within the last 3 months. However, no significant changes on MSOME criteria or WHO criteria in correlation to exercise (frequency, kind and, time of exercises) or sauna could be determined (data not shown).

### Multifactorial impact of life-style factors on semen quality

It has to be kept in mind that all these reported factors do not influence sperm quality each by itself. Among lifestyle factors, all the above mentioned factors have been studied extensively, but the compounding effects are difficult to separate, because they are common lifestyle behaviors. In fact, environmental and personal factors may result in a cumulative outcome. On the other hand, factors which might be crucial for the outcome alone might be obliterated by a multifactorial lifestyle influence. We therefore designed a point-based system to acknowledge this complexity. We scored BMI >25, age >50, coffee intake >3 cups per day, sexual abstinence (>2 days), ejaculation frequency (<4x month) with one point each. Cut-off for ‘unhealthy’ lifestyle was set at more than 2 points. A total number of 1654 patients answered all these questions, 1484 were classed as ‘healthy’ and 170 as ‘unhealthy’ lifestyle patients (Table 5).

**Table 5:** Scoring of patient’s multifactorial lifestyle by impact points in healthy and unhealthy lifestyle groups

	**Total number**	**Healthy lifestyle (< 3 Points)**^**a**^		**Unhealthy lifestyle (> 2 Points)**^**b**^		**p-value**
*Number of Patients*	1654	1484		170		
*ml/sample (mean ± sd)*		2.5	± 1.3	2.8	± 1.6	<0.001
*Mio/ml (mean ± sd)*		23.9	± 32.7	22.4	± 23	n.s.
*Mio/sample (mean ± sd)*		58.5	± 110.2	58.6	± 68.7	n.s.
*Grade a Sperm (%)*		5.3	± 8.2	4.5	± 7.7	<0.05
*Grade b Sperm (%)*		32.5	± 17.9	31.4	± 17.5	n.s.
*Grade c Sperm (%)*		18.3	± 14.4	17.7	± 13.7	n.s.
*Grade d Sperm (%)*		43.9	± 20.4	46.2	± 20.9	n.s.
*Class I (%)*		6.5	± 6.6	5.6	± 5.9	<0.01
*Class II (%)*		42.8	± 14.4	43.7	± 14.4	n.s.
*Class III (%)*		50.7	± 18.2	50.7	± 17.5	n.s.

Factors which might negatively affect sperm quality such as BMI (> 25 kg/m^2^), age (> 50 years), coffee (> 3 cups/day), sexual abstinence (> 2 days) and ejaculation frequency (< 4 times/month) were scored with one point and set in correlation to semen parameters. Cut-off for (un-)healthy lifestyle was set to 2 points. ^a^ 216 patients = 0 points, 656 patients = 1 point; 613 patients = 2 points; ^b^ 149 patients = 3 points; 20 patients = 4 points, 1 patient = 5 points; n.s.: not significant.

We saw a significant (p<0.05) lower sperm quality according to MSOME criteria (class I) and in motility (grade a) in the 'unhealthy' patient subpopulation even though the percentage of class I and grade a sperms were above the average of the total patients.

## Discussion

In order to improve the ART outcome, the influence of certain lifestyle factors on gametes and gamete morphology becomes more and more acknowledged. Many studies show that nutritional habits, smoking and social behavior as well as the environment can affect sperm count, motility and morphology (WHO criteria). Although numerous studies demonstrated the influence of nutrition, lifestyle, age or social behavior on sperm quality, so far - to our knowledge - hardly any study considered the influence of these factors on the rate of vacuolization in the sperm head (MSOME criteria).

Schnall was the first to describe nuclear vacuoles in human spermatozoa as small, non-membrane bound cavities of irregular outline distributed at random throughout the condensed chromatin in 1952 [45].

Even though the origin of vacuoles is still not completely understood and it has been fiercely discussed whether those vacuoles originate from a natural process or, more likely, from pathological (stress) situations during spermatogenesis [21,22], a study by Brassesco and colleagues demonstrated that sperm vacuolization improves after antioxidant therapy [46]. This confirms speculations that oxidative stress might play a major role in these processes and the imbalance between reactive oxygen species (ROS) and the cells antioxidative response might be one of the main causes for male infertility beside genetic reasons. Especially in ART patients, higher rates of sperm with extensive vacuolization are found, which might contribute to a lower IVF outcome [12].

Therefore antioxidants were widely believed to improve sperm quality but are still the subject of a controversial debate. Numerous studies based on animal model systems as well as on humans depict the benefit of antioxidative and micronutrient supplementation on semen quality [47-52]. This is in concordance with our earlier findings that intake of micronutrient and antioxidative supplement (Fertilovit® M^plus^) significantly improved semen parameters of 160 patients even under MSOME criteria (unpublished observations). Additionally, based on our findings that the stated fruit/vegetable intake is remarkably low (1.3 portions / day), we recommend a general antioxidative supplementation. The Joint FAO/WHO Expert Consultation on diet, nutrition and the prevention of chronic diseases recommend the intake of a minimum of 400g of fruit and vegetables per day (>= 3–5 servings).

Even though certain sperm selection methods such as MSOME have greatly improved outcomes of fertility treatment, it might be eligible to ameliorate the gamete quality. According to this study, protective factors such as healthy nutrition, frequent ejaculations and short periods of abstinence seem to be capable of contributing to this amelioration. The effect of detrimental factors such as aging, increased BMI, high coffee and nicotine intake can eventually be reversed by these protective impact factors. The impact of psychological stress on sperm quality remains under debate.

In summary, our study displays the influence of numerous lifestyle factors on sperm parameters regarding both WHO and MSOME criteria in single and multifactorial manner. According to the literature, most of the adverse health factors such as smoking, obesity, age, malnutrition or infection of genital tract effects were linked to elevated oxidative stress levels or an inadequate quenching of ROS [47]. Moreover, as it was recently demonstrated that antioxidative supplementation can improve sperm DNA in men of an advanced age [52]. Whether the grade of vacuolization is directly affected by oxidative stress remains questionable but in light of our own studies, we suppose that this is the case.

Nevertheless, further studies will be needed to prove whether antioxidative supplementation can overcome a combination of lifestyle factors having a negative impact on sperm quality.

## Conclusions

In conclusion, the present results reveal a significant decline of sperm morphology (MSOME) by the additive impact of advanced age and detrimental lifestyle factors such as high BMI, low sexual activity and high coffee intake. To our knowledge, this is the first time that such a correlation of lifestyle factors and MSOME quality was presented. Moreover, we here demonstrated that not only unique lifestyle factor(s) impact on sperm morphology but distinct factors act in a cummulative way. Considering the relationship between nuclear vacuoles and decreased IVF outcome in terms of poor embryo development or unsuccessful pregnancy it is mandatory to consult patients about their lifestyle behavior and to advise them to reconsider their lifestyle in order to improve their IVF chances.

## Abbreviations

ART: Assisted reproductive technologies; BMI: Body-mass index; FAO: Food and Agriculture Organization of the United Nations; ICSI: Intracytoplasmic Sperm Injection; IMSI: Intracytoplasmic Morphologically Selected Sperm Injection; IVF: In vitro fertilization; MSOME: Motile sperm organelle morphology examination); ROS: Reactive oxygen species; TSC: Total sperm count; WHO: World Health Organization.

## Competing interests

The authors declare that they have no competing interests.

## Authors' contributions

JW and NHZ designed and coordinated the study. All authors were responsible for the data collection, analysis, and interpretation presented in the manuscript. BW performed the statistical analyses. MS and BS wrote the manuscript. AS, PV and BW reviewed the manuscript. All authors read and approved the final manuscript.

## Supplementary Material

Additional file 1 **Supplemental Table S1.** Drug intake of patient. Click here for file

## References

[B1] Braga DP Halpern G Figueira Rde C Setti AS Iaconelli A Jr Borges E Jr Food intake and social habits in male patients and its relationship to intracytoplasmic sperm injection outcomes Fertil Steril 2012 97 53 59 10.1016/j.fertnstert.2011.10.011 22078783

[B2] Sekhavat L Moein MR The effect of male body mass index on sperm parameters Aging Male 2010 13 155 158 10.3109/13685530903536643 20059435

[B3] Gaur DS Talekar MS Pathak VP Alcohol intake and cigarette smoking: impact of two major lifestyle factors on male fertility Indian J Pathol Microbiol 2010 53 35 40 10.4103/0377-4929.59180 20090219

[B4] Jensen TK Swan SH Skakkebaek NE Rasmussen S Jørgensen N Caffeine intake and semen quality in a population of 2,554 young danish men Am J Epidemiol 2010 171 883 891 10.1093/aje/kwq007 20338976

[B5] Jensen TK Andersson AM Jørgensen N Andersen AG Carlsen E Petersen JH Skakkebaek NE Body mass index in relation to semen quality and reproductive hormones among 1,558 danish men Fertil Steril 2004 82 863 870 10.1016/j.fertnstert.2004.03.056 15482761

[B6] Kruger TF Acosta AA Simmons KF Swanson RJ Matta JF Veeck LL Morshedi M Brugo S New method of evaluating sperm morphology with predictive value for human in vitro fertilization Arch Androl 1987 18 275 277 10.3109/01485018708988493 3629768

[B7] Menkveld R Kruger TF Advantages of strict (tygerberg) criteria for evaluation of sperm morphology Int J Androl 1995 18 36 42 Review 8719857

[B8] World Health Organization WHO Laboratory Manual for the Examination of Human Semen and Sperm-cervical Mucus Interaction 1992 3 Cambridge University Press, Cambridge 107p

[B9] World Health Organisation Department of Reproductive Health and Research WHO laboratory manual for the examination and processing of human semen 2010 5 287p

[B10] Bartoov B Berkovitz A Eltes F Kogosowski A Menezo Y Barak Y Real-time fine morphology of motile human sperm cells is associated with IVF-ICSI outcome J Androl 2002 23 1 8 11780915 10.1002/j.1939-4640.2002.tb02595.x

[B11] Berkovitz A Eltes F Soffer Y Zabludovsky N Beyth Y Farhi J Levran D Bartoov B ART success and in vivo sperm cell selection depend on the ultramorphological status of spermatozoa Andrologia 1999 31 1 8 9949882

[B12] Vanderzwalmen P Hiemer A Rubner P Bach M Neyer A Stecher A Uher P Zintz M Lejeune B Vanderzwalmen S Blastocyst development after sperm selection at high magnification is associated with size and number of nuclear vacuoles Reprod Biomed Online 2008 17 617 627 10.1016/S1472-6483(10)60308-2 18983745

[B13] Berkovitz A Eltes F Lederman H Peer S Ellenbogen A Feldberg B Bartoov B How to improve IVF-ICSI outcome by sperm selection Reprod Biomed Online 2006 12 634 638 10.1016/S1472-6483(10)61191-1 16790113

[B14] Hazout A Dumont-Hassan M Junca AM Cohen Bacrie P Tesarik J High-magnification ICSI overcomes paternal effect resistant to conventional ICSI Reprod Biomed Online 2006 12 19 25 16454928 10.1016/s1472-6483(10)60975-3

[B15] Antinori M Licata E Dani G Cerusico F Versaci C d'Angelo D Antinori S Intracytoplasmic morphologically selected sperm injection: a prospective randomized trial Reprod Biomed Online 2008 16 835 841 10.1016/S1472-6483(10)60150-2 18549694

[B16] Nadalini M Tarozzi N Distratis V Scaravelli G Borini A Impact of intracytoplasmic morphologically selected sperm injection on assisted reproduction outcome: a review Reprod Biomed Online 2009 19 45 55 Review 20034423 10.1016/s1472-6483(10)60283-0

[B17] Souza Setti A Ferreira RC Paes de Almeida Ferreira Braga D de Cássia Sávio Figueira R Iaconelli A Jr Borges E Jr Intracytoplasmic sperm injection outcome versus intracytoplasmic morphologically selected sperm injection outcome: a meta-analysis Reprod Biomed Online 2010 21 450 455 Review 10.1016/j.rbmo.2010.05.017 20800549

[B18] Knez K Zorn B Tomazevic T Vrtacnik-Bokal E Virant-Klun I The IMSI procedure improves poor embryo development in the same infertile couples with poor semen quality: a comparative prospective randomized study Reprod Biol Endocrinol 2011 9 123 10.1186/1477-7827-9-123 21875440 PMC3170257

[B19] Oliveira JB Petersen CG Massaro FC Baruffi RL Mauri AL Silva LF Ricci J Franco JG Jr Motile sperm organelle morphology examination (MSOME): intervariation study of normal sperm and sperm with large nuclear vacuoles Reprod Biol Endocrinol 2010 8 56 10.1186/1477-7827-8-56 20529256 PMC2889997

[B20] Hammoud I Boitrelle F Ferfouri F Vialard F Bergere M Wainer B Bailly M Albert M Selva J Selection of normal spermatozoa with a vacuole-free head (x6300) improves selection of spermatozoa with intact DNA in patients with high sperm DNA fragmentation rates Andrologia 2012 26 10.1111/j.1439-0272.2012.01328.x 22731614

[B21] Boitrelle F Ferfouri F Petit JM Segretain D Tourain C Bergere M Bailly M Vialard F Albert M Selva J Large human sperm vacuoles observed in motile spermatozoa under high magnification: nuclear thumbprints linked to failure of chromatin condensation Hum Reprod 2011 26 1650 1658 10.1093/humrep/der129 21536591

[B22] Franco JG Jr Mauri AL Petersen CG Massaro FC Silva LF Felipe V Cavagna M Pontes A Baruffi RL Oliveira JB Large nuclear vacuoles are indicative of abnormal chromatin packaging in human spermatozoa Int J Androl 2012 35 46 51 10.1111/j.1365-2605.2011.01154.x 21535011

[B23] Skowronek F Casanova G Alciaturi J Capurro A Cantu L Montes JM Sapiro R DNA sperm damage correlates with nuclear ultrastructural sperm defects in teratozoospermic men Andrologia 2012 44 59 65 10.1111/j.1439-0272.2010.01106.x 21592172

[B24] Vanderzwalmen P Bertin-Segal G Geerts L Debauche C Schoysman R Sperm morphology and IVF pregnancy rate: comparison between percoll gradient centrifugation and swim-up procedures Hum Reprod 1991 6 581 588 1918311 10.1093/oxfordjournals.humrep.a137383

[B25] Tesarik J Greco E Mendoza C Late, but not early, paternal effect on human embryo development is related to sperm DNA fragmentation Hum Reprod 2004 19 611 615 10.1093/humrep/deh127 14998960

[B26] Zini A Libman J Sperm DNA damage: importance in the era of assisted reproduction CMAJ 2006 175 495 500 Review 10.1503/cmaj.060218 16940270 PMC1550758

[B27] World Health Organization Obesity: preventing and managing the global epidemic. Report of a WHO consultation World Health Organ Tech Rep Ser 2000 894 1 253 11234459

[B28] Aggerholm AS Thulstrup AM Toft G Ramlau-Hansen CH Bonde JP Is overweight a risk factor for reduced semen quality and altered serum sex hormone profile? Fertil Steril 2008 90 619 626 10.1016/j.fertnstert.2007.07.1292 18068160

[B29] Wegner CC Clifford AL Jilbert PM Henry MA Gentry WL Abnormally high body mass index and tobacco use are associated with poor sperm quality as revealed by reduced sperm binding to hyaluronan-coated slides Fertil Steril 2010 93 332 334 10.1016/j.fertnstert.2009.07.970 19733846

[B30] Qin DD Yuan W Zhou WJ Cui YQ Wu JQ Gao ES Do reproductive hormones explain the association between body mass index and semen quality? Asian J Androl 2007 9 827 834 10.1111/j.1745-7262.2007.00268.x 17968470

[B31] Silva LF Oliveira JB Petersen CG Mauri AL Massaro FC Cavagna M Baruffi RL Franco JG Jr The effects of male age on sperm analysis by motile sperm organelle morphology examination (MSOME) Reprod Biol Endocrinol 2012 19 10 19 10.1186/1477-7827-10-19 PMC3317862 22429861

[B32] Hubert A Szöke A Leboyer M Schürhoff F Influence of paternal age in schizophrenia Encéphale 2011 37 199 206 Review 21703435 10.1016/j.encep.2010.12.005

[B33] Reichenberg A Gross R Sandin S Susser ES Advancing paternal and maternal age are both important for autism risk Am J Public Health 2010 100 772 773 10.2105/AJPH.2009.187708 20299637 PMC2853632

[B34] Frans EM Sandin S Reichenberg A Lichtenstein P Långström N Hultman CM Advancing paternal age and bipolar disorder Arch Gen Psychiatry 2008 65 1034 1040 10.1001/archpsyc.65.9.1034 18762589

[B35] Saha S Barnett AG Foldi C Burne TH Eyles DW Buka SL McGrath JJ Advanced paternal age is associated with impaired neurocognitive outcomes during infancy and childhood PLoS Med 2009 6 e40 10.1371/journal.pmed.1000040 19278291 PMC2653549

[B36] Oldereid NB Rui H Purvis K Life styles of men in barren couples and their relationship to sperm quality Int J Fertil 1992 37 343 349 1360454

[B37] Mitra A Chakraborty B Mukhopadhay D Pal M Mukherjee S Banerjee S Chaudhuri K Effect of smoking on semen quality, FSH, testosterone level, and CAG repeat length in androgen receptor gene of infertile men in an indian city Syst Biol Reprod Med 2012 58 255 62 10.3109/19396368.2012.684195 22578234

[B38] Selit I Basha M Maraee A El-Naby SH Nazeef N El-Mehrath R Mostafa T Sperm DNA and RNA abnormalities in fertile and oligoasthenoteratozoospermic smokers Andrologia 2012 10.1111/j.1439-0272.2012.01305.x in press 22564026

[B39] Saleh RA Agarwal A Sharma RK Nelson DR Thomas AJ Jr Effect of cigarette smoking on levels of seminal oxidative stress in infertile men: a prospective study Fertil Steril 2002 78 491 499 10.1016/S0015-0282(02)03294-6 12215323

[B40] Hjollund NH Bonde JP Henriksen TB Giwercman A Olsen J Danish first pregnancy planner study team. Reproductive effects of male psychologic stress Epidemiology 2004 15 21 27 10.1097/01.ede.0000100289.82156.8b 14712143

[B41] Hjollund NH Bonde JP Henriksen TB Giwercman A Olsen J Danish first pregnancy planner study team. Job strain and male fertility Epidemiology 2004 15 114 117 10.1097/01.ede.0000100290.90888.4a 14712155

[B42] Eskiocak S Gozen AS Taskiran A Kilic AS Eskiocak M Gulen S Association between mental stress & some antioxidant enzymes of seminal plasma Indian J Med Res 2005 122 491 496 16517999

[B43] Eskiocak S Gozen AS Kilic AS Molla S Effect of psychological stress on the L-arginine-nitric oxide pathway and semen quality Braz J Med Biol Res 2006 39 581 588 10.1590/S0100-879X2006000500003 16648894

[B44] Kennaway DJ Boden MJ Varcoe TJ Circadian rhythms and fertility Mol Cell Endocrinol 2012 349 56 61 10.1016/j.mce.2011.08.013 21872642

[B45] Schnall MD Electronmicroscopic study of human spermatozoa Fertil Steril 1952 3 62 82 14906383 10.1016/s0015-0282(16)30785-3

[B46] Brassesco M Lopez G Monqaut A Lafuente R Sperm vacuole improvement after antioxidant therapy Fertil Steril 2011 96 159

[B47] Makker K Agarwal A Sharma R Oxidative stress & male infertility Indian J Med Res 2009 129 357 367 Review 19535829

[B48] Showell MG Brown J Yazdani A Stankiewicz MT Hart RJ Antioxidants formale subfertility Cochrane Database Syst Rev 2011 1 CD007411 Review 10.1002/14651858.CD007411.pub2 21249690

[B49] Mistry HD Broughton Pipkin F Redman CW Poston L Selenium in reproductive health Am J Obstet Gynecol 2012 206 21 30 Review 10.1016/j.ajog.2011.07.034 21963101

[B50] Moslemi MK Tavanbakhsh S Selenium-vitamin E supplementation in infertile men: effects on semen parameters and pregnancy rate Int J Gen Med 2011 4 99 104 21403799 10.2147/IJGM.S16275 PMC3048346

[B51] Oliva A Dotta A Multigner L Pentoxifylline and antioxidants improve sperm quality in male patients with varicocele Fertil Steril 2009 91 1536 1539 10.1016/j.fertnstert.2008.09.024 18990381

[B52] Schmid TE Eskenazi B Marchetti F Young S Weldon RH Baumgartner A Anderson D Wyrobek AJ Micronutrients intake is associated with improved sperm DNA quality in older men Fertil Steril 2012 98 1130 1137 10.1016/j.fertnstert.2012.07.1126 22935557

