# Supplementary material for: The combination matters - distinct impact of lifestyle factors on sperm quality: a study on semen analysis of 1683 patients according to MSOME criteria
Source: Reprod Biol Endocrinol. 2012 Dec 24;10:115. doi: 10.1186/1477-7827-10-115 (PMC3575231; doi:10.1186/1477-7827-10-115)
Supplement: Additional file 1 — Supplemental Table S1. Drug intake of patient. [file 1477-7827-10-115-S1.doc]

**Supplemental Table S1. Drug intake of patient**

| **Kind of pharmaceuticals intake** | **Number of patients ^a^** |
| --- | --- |
| *antibiotics* | 31 |
| *antihypertensives* | 16 |
| *beta-adrenergic blocking agents* | 5 |
| *coagulation drugs* | 11 |
| *cholesterol lowering medications* | 7 |
| *corticoids* | 4 |
| *painkillers* | 55 |
| *proton pump inhibitors* | 5 |
| *thyroid drugs* | 8 |
| *5-alpha reductase inhibitor* | 3 |
| *not declared* | 27 |
| *others* | 284 |

^a^ multiple answers were permitted
